# Grounding verbs in action: The facilitative effect of potential physical interactions with verbs

**DOI:** 10.3758/s13423-025-02776-5

**Published:** 2026-01-05

**Authors:** Devin Dickinson, Mark Yates

**Affiliations:** Psychology Department, University of South Alabama, 75 S. University Blvd., Mobile, AL 36688 USA

**Keywords:** Semantics, Word recognition, Embodied cognition

## Abstract

Grounded cognition holds that a word’s semantic representation is grounded in sensory-motor experiences. Much research has supported this account in relation to nouns, but less is known about verbs. The current research was designed to test the effect of grounded cognition with relation to action verbs. Accordingly, we developed a measure labeled potential physical interactions (PPI) aimed at capturing grounded content by measuring the number of physical objects an action verb can interact with. We first collected PPI ratings from 177 university students. Following this, a different group of 40 university students made lexical decisions to the words. The results indicate that words higher in PPI are responded to more rapidly than are those lower in PPI. We replicated this finding with data from the English Lexicon Project. The findings support the claim that the semantic representation of action verbs is grounded in sensory-motor experience.

**Supplementary Information:**

The online version contains supplementary material available at 10.3758/s13423-025-02776-5.

## Introduction

Language is central to the human experience and as such is relevant to many areas of psychology. One of the most debated topics in language research is the nature of the semantic representation. There have been many variables studied in terms of defining semantics, and collectively the results seem to indicate that words with richer semantic representations are processed more rapidly than are those with less rich representations (Pexman, 2012, 2020). Semantic richness refers to the amount and variety of semantic information associated with a word. Those that are considered semantically rich have more connections, features, meanings, or contexts. This richness usually leads to faster and more accurate responses in word-recognition tasks. Below we give a brief overview of some of the previous work on semantic richness before turning to the current work on grounded representations.

One way of defining meaning is in terms of semantic features where semantic features are a count of the distinct conceptual attributes related to a word. As an example, *knife* has as some of its features “has a handle,” “is sharp,” and “is used for cutting” (McRae et al., 2005). Research has shown that words with more semantic features are processed more rapidly (McRae et al., 2005; Pexman et al., 2002, 2003).

Semantic neighborhood density is a measure that refers to the number of semantically related words (e.g., from word-association norms or co-occurrence models) that are closely linked to the target word. Some of the semantic neighbors of *knife* include *fork, stab,* and *gun* (Nelson et al., 2004). Words with denser semantic neighborhoods have shown faster lexical decision latencies (Buchanan et al., 2001; Locker et al., 2003; although see Mirman & Magnuson, 2008).

Semantic diversity is a variable that assesses the variability in written linguistic contexts in which a word appears. The more disparate the contexts the larger a word’s semantic diversity. This variable has similarly shown decreased reading time for words with more contexts (Hoffman et al., 2013; Hoffman & Woollams, 2015).

In general, studies on semantic richness, regardless of how it is defined, usually show that more features, neighbors, or contexts facilitate processing. Given as such, models of word recognition need to account for how increased semantic activation facilitates lexical processing. To this end, some have argued that semantic activation feeds back to the orthographic level to support lexical decisions (Pexman et al., 2002; Yap et al., 2015). This feedback explanation provides a theoretical framework in which new semantic effects can be explained and will be central to our account of the potential physical interactions (PPI) measure we introduce below.

Before introducing the PPI measure, we review the work on grounded cognition that serves as the theoretical framework for PPI. Grounded accounts propose that word meanings are rooted in our perceptual and motor experience. In this view, sensory and motor information play a central role in constructing meaning via an experiential, modal format (Barsalou, 2008; Barsalou et al., 2003; Glenberg, 2015). Contrasted with an amodal format that frames semantic information by means of symbols or feature lists, the modal format uses the full range of experience for defining semantic information (Lakoff, 2012). Theories using grounded cognition, such as Barsalou’s (1999) Perceptual Symbol Systems, further explain how sensory-motor experience is key to defining semantics. This framework contrasts with the semantic variables discussed earlier. Both semantic neighborhood and semantic diversity are distributional measures that arise from the usage-based theories of semantic memory. Semantic features are traditionally amodal, representing conceptual properties in a symbolic format. That said, many feature norms include perceptual and motor attributes, making them theoretically compatible to some extent with grounded accounts of meaning.

Under the grounded cognition hypothesis, sensory-motor information has demonstrated a key role in the process of lexical retrieval. Effects such as the body-object interaction (BOI; Siakaluk et al., 2008) and sensorimotor strength (Lynott et al., 2020) provide findings congruent with theories of grounded cognition. Both effects demonstrate the ability for sensory-motor information to affect lexical processes in significant ways across words.

BOI effects have shown that when considering the manipulability of an object, a word’s referent provides faster reaction times when highly manipulable. For example, when considering the object *bottle*, being able to manipulate this object through actions such as grabbing it, throwing it, and so on, provides semantic content for lexical processes and in turn facilitation for lexical retrieval. This ultimately suggests that when richer motor information is available, it facilitates semantic feedback effects.

Similarly, norms collected by Lynott et al. (2020) have shown that the strength of both the perceptual and the action components of a word are important determinates of word recognition latencies. These norms consider more specific sensorimotor aspects unique to a given word across a broad range of stimuli constituting its sensorimotor strength. From these more fine-tuned measures of sensorimotor interaction with perceptual and action as a basis for a given word, their results imply that more semantic information facilitates word recognition. Typically, studies on variables of semantic richness have focused on collecting measures regarding noun stimuli (Amsel et al., 2012; Yap et al., 2012, 2015). However, recent studies have observed semantic richness effects on verb stimuli to more specifically test hypotheses of grounded cognition given its predictions on action-oriented experience in relation to meaning. In terms of what the hypothesis of grounded cognition proposes, actions and their meanings should be more wholly encapsulated by experiences and the re-emulation of the specific contexts and sensory-motor features of those experiences (Barsalou et al., 2003). Given such, verbs and their potential semantic processes should demonstrate effects similar to nouns by way of semantic richness effects.

In terms of results found with verbs, Sidhu et al. (2014) demonstrated facilitated response times with a variable they labeled Relative Embodiment (RE), which measures the relative sense of body perception in relation to verbs. As with the previous semantic richness effects on BOI and sensorimotor strength, those experiences that generate more semantic information facilitate lexical recognition, and with the RE measure, the relative sense of body experience the verb evokes serves as a metric of the semantic richness of the verb under question. For example, verbs such as *dance* or *breathe* describe more grounded bodily experiences and as such rate higher in RE and produce faster reaction times. In contrast, verbs such as *evaporate* and *expect* are not as grounded in bodily experience, thus rate lower in RE and are slower to be recognized.

It seems then that verbs and their semantic content may work in a way similar to how BOI and sensorimotor strength influence nouns such that verbs that evoke more content for concrete action experiences have richer semantic representations and are recognized more rapidly. As such, comparable results for verbs also indicate that their semantic content may work in a manner congruent with hypotheses of grounded cognition accounts on noun stimuli and that other potential measures of verb meaning could produce findings in line with those on nouns. Given such, as predicted with semantic richness effects with variables such as BOI and sensorimotor strength, as well as findings with RE, we introduce a new construct, *potential physical interactions (PPI)*, which indexes the number of distinct objects a verb can plausibly interact with.

PPI as a measure denotes features of verbs by way of evaluating the number of objects verbs can interact with. We hypothesized in line with other semantic richness variables that verbs that elicited high PPI ratings would be processed more rapidly in the lexical decision task. Verbs high in PPI should have richer semantic representations and, thereby, facilitate semantic processing. This semantic activation was expected to feed back to the orthographic level and support lexical decisions. For example, the verb *drop* would presumably have many interactable objects (e.g., a cup, an apple, a ball, etc.), whereas the verb *knit* should have fewer objects (e.g., needles, yarn, etc.). These should be reflected in the ratings with words like *drop* being rated higher in PPI and responded to more rapidly in the lexical decision task than words like *knit*, which should be rated lower and recognized more slowly.

We interpret PPI as indexing the breadth of a verb’s semantic representation in terms of potential grounded interactions. A verb like *drop* can apply to a wide range of objects, yielding a representation that is more distributed and reflects broad interaction patterns across many contexts. High PPI verbs should engage perceptual and motor systems in a flexible manner, supporting rapid word recognition. In contrast, verbs such as *knit* are tied to narrower, more object-specific experiences, requiring activation of detailed motor routines and perceptual information associated with specific objects (e.g., yarn, needles). From this perspective, PPI captures the range of grounded interactions available to a verb. Verbs with broader representations (high PPI) are easier to process in lexical decision because they activate a larger set of potential interactions, whereas verbs with narrower representations (low PPI) are more constrained.

The focus of the current study is to examine the effect of PPI in a lexical decision task as a means of testing whether verb semantic representations are grounded in perceptual experiences and motor actions as predicted by the grounded cognition hypothesis. It also allows us to test effects of semantic richness in relation to verbs using a measure that captures unique aspects of verbs not captured by other verb measures like RE.

In line with previous work on semantic richness variables such as BOI, sensorimotor strength, and RE (Bennett et al., 2011; Siakaluk et al., 2008; Sidhu et al., 2014), our hypothesis for the current work was that words having richer semantic representation as indexed by higher PPI ratings will be responded to more rapidly in the lexical decision task than to those words with less rich semantic representations. We tested this using two different data sets. First, we collected lexical decisions from participants and used a linear mixed effect model to test the hypothesis. Next, we used the reaction times from the English Lexicon Project (ELP; Balota et al., 2007) and fit a linear regression model using the PPI ratings and same control variables as in the mixed effects model.

## Methods

### Participants

#### Potential physical interaction (PPI) ratings

The current set of stimuli included 100 single-syllable verbs and 98 two-syllable verbs and were rated by 205 University of South Alabama students who received course credit. All participants were asked to rate the target verbs on a 1 (few) to 7 (many) scale. On this scale, a “many” PPI rating would indicate a large list of potential interactions for a verb, and a “few” rating would indicate a small number of potential interactions for a verb (see Appendix in the Online Supplementary Material for instructions). All participants were native English speakers. A total of 28 (remaining *n* = 177) participants were removed from the data set as they had more than 10% missing responses. Verbs were presented in infinitive form (e.g., *to break*).

#### Lexical decision task

A different group of 44 students were recruited for the lexical decision task through the subject pool at the University of South Alabama's psychology department. Participants were granted course credit for their participation. Participants included in the final analysis met the requirements of being native English speakers, having normal or corrected-to-normal vision, and not being diagnosed with a reading disability. Four subjects were removed for self-reporting a diagnosis of dyslexia, resulting in a total of 40 participants being included in the analysis.

### Stimuli

Initial experimental stimuli consisted of 198 action verbs for which we collected ratings. However, two were inadvertently not included in the lexical decision task, these being *contract* and *juggle*. Only 196 final words from the original stimuli set were included in the lexical decision task. The verbs were presented in lemma form (e.g., *break*). The nonword distractors consisted of 196 pseudohomophones that were intermixed with the verb stimuli. All pseudohomophones for the experiment are matched in length to the verbs. Similarly, ratings such as AoA, frequency, BOI, length, concreteness, phonological neighborhood, and orthographic neighborhood were gathered through the ELP database (Balota et al., 2007) to use as control variables in the analyses.

### Procedure

Subjects participated in a lexical decision task coded in Eprime2 (Schneider et al., 2002). Instructions were given to the participants to respond as quickly and accurately as possible to the stimuli presented. The participants were given ten practice trials before the experimental trials. None of these practice stimuli were used as experimental stimuli. Stimulus order was randomized for each participant. Each letter string was presented after a 1,000-ms fixation mark (+). Participants made their responses with their right hand, which was placed on the number pad of the keyboard. They pressed the Num1 key with their index finger for word responses and the Num2 key with their middle finger for nonword responses. Reaction times were measured between presentation of the stimulus and the following response.

## Results

### PPI descriptives

**Fig. 1:**
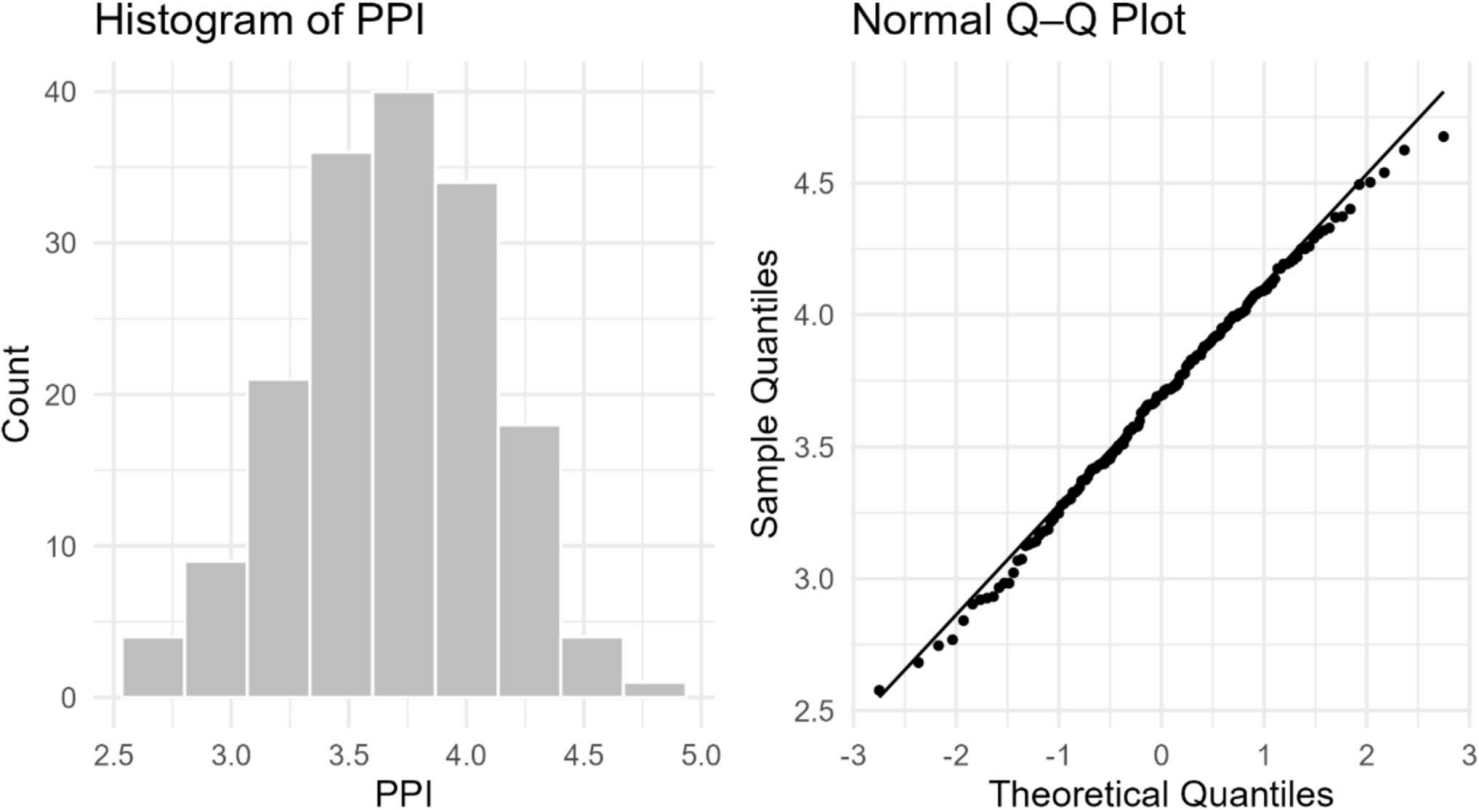
Histogram and QQ plot of potential physical interactions (PPI)

Before presenting the results from the reaction time analyses, we first provide descriptive measures of the PPI ratings from the words used in the linear mixed-effects models (LMMs) below. Figure 1 provides a histogram and QQ plot for PPI. Visual inspection indicates the distribution is roughly normal. This is congruent with the descriptive statistics in Table 1.

**Table 1:** Descriptive statistics for potential physical interactions (PPI)

Statistic	Value
Mean	3.68
SD	0.42
Skew	−0.15
SE (Skew)	0.19
Kurtosis	−0.26
SE (Kurtosis)	0.37

### Reaction time analyses

In the lexical decision task, only reaction times to correct responses to words were used in the analysis. Any responses generated by participants that were less than 250 ms or greater than 2,000 ms were removed from the data (0.012% of cases). Similarly, items that had accuracy rates of less than 50%, as well as subjects with accuracy less than 80%, were removed from the data. Based on these criteria, two items (*congeal* and *nab*) and three subjects were removed (*n* = 37). In the final analysis, 27 words did not have BOI or other values for the fixed effects in the ELP, and thus were not included in the final model, with a resulting final set consisting of 167 words.

For the analysis, an LMM was used to test the effect of PPI, with the other variables included as fixed effects as well. This allows us to test the PPI effect while holding the other variables constant in the model. In the model, random intercepts for subjects and items were put in, as well as by subject random slopes for PPI. We applied the inverse transformation (1/RT) then multiplied by ˗1,000 leading to ˗1,000/RT. Multiplying by ˗1,000 removes the decimals and reflects the inverse reaction times so that larger inverse reaction times indicate slower responding as is the case for raw reaction times. The subsequent model was used in the final analysis:

$$InverseRT\sim Inverse\;Previous\;RT+Age\;of\;Acquisition+Body\;Object\;Interaction+Concreteness\;Rating+Length+Log\;Frequency+Orthographic\;Neighborhood+Phonological\;Neighborhood+PPI+\left(1+PPI\vert subject\right)+\left(1\vert item\right)$$

This model considers age of acquisition, body object interaction, concreteness ratings, word length, log frequency, orthographic neighborhood, phonological neighborhood, and PPI as fixed effects. The model also includes inverse previous reaction times as a fixed effect, as reaction times to the previous item have been found to significantly affect the next item presented (Baayen & Milin, 2010). To fit this effect in the model, all first trials for each participant were removed, as they do not have a previous reaction time to measure. The correlations for all variables and average reaction times can be found in Table 2.

**Table 2:** Means, standard deviations, and correlations of lexical variables

Variable	Means (*SD*)	1	2	3	4	5	6	7	8	9
1. PPI	3.68 (0.42)	--								
2. Length	5.22 (1.29)	−0.26**	--							
3. Freq	2.68 (0.73)	0.71**	−0.46**	--						
4. Ortho.	5.63 (5.61)	0.24**	−0.71**	0.40**	--					
5. Phono.	13.50 (14.34)	0.31**	−0.64**	0.48**	0.71**	--				
6. Conc.	3.82 (0.61)	−0.06	−0.30**	0.16	0.33**	0.31**	--			
7. AoA	7.04 (2.11)	−0.56**	0.49**	−0.64**	−0.42**	−0.45**	−0.40**	--		
8. BOI	3.03 (1.09)	−0.08	−0.21*	0.17	0.23*	0.22**	0.69**	−0.26**	--	
9. RT	669.42 (67.19)	−0.65**	0.24*	−0.65**	−0.19	−0.27**	−0.15	0.56**	−0.16	--

** p < 0.001, * p < 0.05

PPI = Potential Physical Interactions, Freq. = frequency, Ortho. = orthographic neighborhood, Phono. = phonological neighborhood, Conc. = concreteness rating, AoA = age of acquisition, BOI = body-object interaction, RT = average reaction time

The analysis of the linear mixed effect model was run in R (version 4.4.1) using the lme4 (version 1.1–35.5.1.5; Bates et al., 2023) and lmerTest (version 3.1-3.1.1; Kuzentsova et al., 2020) packages to examine the output of the LMM model specified. The lmerTest package provided both Satterthwaite-adjusted degrees of freedom and p-values, with p-values < 0.05 being considered statistically significant. The model was initially fit and any cases with absolute standardized residuals exceeding 2.5 standard deviations were removed (Baayen & Milin, 2010). This resulted in the removal of 2.2% of the data. The model was run again with the removed outliers. All fixed-effect variables were Z-scored except for inverse previous reaction times. The output of parameter estimates, standard errors, t-values, degrees of freedom, and p-values are provided in Table 3. The PPI ratings, data, and R Markdown file for the models are located on the OSF site listed in the *Declarations* section.^1^

**Table 3:** Linear mixed model estimates of fixed effects

Variables	Estimate	SE	T	df	*p*-value
(Intercept)	−1.459	0.032	−46.31	56.99	p < 0.001
Inverse Prev. RT	0.092	0.009	9.800	5653	p < 0.001
AoA	0.026	0.009	2.702	152.1	0.008
BOI	−0.009	0.009	−1.075	153.4	0.284
Concreteness	−0.003	0.01	−0.365	154.4	0.716
Length	0.001	0.01	0.057	154.8	0.954
Freq.	−0.042	0.01	−4.060	154.6	*p* < 0.001
Ortho. N.	0.017	0.01	1.680	153.2	0.095
Phono. N.	−0.001	0.01	−0.097	152.3	0.923
PPI	−0.043	0.01	−4.154	142.9	*p* < 0.001

AoA = age of acquisition, BOI = body-object interaction, Freq. = frequency, Ortho. N. = orthographic neighborhood, Phono. N. = phonological neighborhood, PPI = potential physical interactions

The results demonstrated that the effects of log frequency, AoA, inverse reaction time, and PPI were all significant predictors of reaction times in the current lexical decision task. Inverse previous reaction time was significant, suggesting that as previous reaction times increased, current trial reaction time increased. Furthermore, there was a frequency effect, showing that subject reaction times decreased when the word had a higher frequency. Moreover, there was an AoA effect, showing that as a word’s AoA rating increased, reaction time increased as well. Importantly, the PPI effect suggests that reaction times decrease as PPI increases. This indicates a clear facilitative effect of PPI as hypothesized.

To replicate the result of the above lexical decision task with another set of latencies, the ELP latencies were gathered and analyzed in a linear regression with the fixed effects in Table 3. The results of the analysis are found in Table 4. We used the standardized reaction times (zRT) from ELP and the unstandardized values for the predictors as is commonly done in psycholinguistic megastudies (e.g., Cortese et al., 2018). A simple regression with the following formula was run for the analysis:

**Table 4:** Regression analysis on English Lexicon Project (ELP) lexical decision latencies

Variables	Estimate	SE	t	*p*-value
(Intercept)	0.060	0.223	0.271	0.787
AoA	0.007	0.008	0.909	0.365
BOI	−0.029	0.014	−1.992	0.048
Concreteness	0.024	0.028	0.842	0.401
Length	0.010	0.013	0.765	0.445
Freq.	−0.137	0.025	−5.382	*p* < 0.001
Ortho. N.	0.001	0.003	0.439	0.662
Phono. N.	−7.598e-5	0.001	−0.064	0.949
PPI	−0.088	0.042	−2.090	0.038

AoA = age of acquisition, BOI = body-object interaction, Freq. = frequency, Ortho. N. = orthographic neighborhood, Phono. N. = phonological neighborhood, PPI = potential physical interactions

$$zRT\sim Age\;of\;Acquisition+Body\;Object\;Interaction+Concreteness\;Rating+Length+Log\;Frequency+Orthographic\;Neighborhood+Phonological\;Neighborhood+PPI$$

The results corroborate the effect of the PPI variable when tested against ELP latencies, showing that higher PPI ratings lead to quicker lexical decisions.

## Discussion

The purpose of the current study was to investigate the effects of the PPI measure in a lexical decision task as a means of testing the grounded cognition account of semantic representations. It also allows another test of the semantic richness effect on visual word recognition. Based on the grounded cognition account, we hypothesized that as PPI increases the semantic content for a verb likewise increases. Following previous research on semantic richness, the result of this increased semantic content should be faster lexical decisions. That is, PPI should facilitate lexical decisions. The results across two data sets unambiguously support this prediction. Given these results, it is clear PPI plays a key role in semantic processing of action verbs.

Given that most of the literature regarding semantic representations and lexical retrieval focus on noun stimuli, the study and confirmation of similar semantic processes regarding verbs seems to confirm that the semantic representation of verbs is also important in their recognition and that their semantic representation is grounded in nature. This follows the findings from Sidhu et al. (2014), who found similar semantic effects on verbs for their RE measure. The results from Sidhu et al. indicated that the degree to which a verb is grounded in the human body is an important determinant of word recognition latencies. The results presented here extend this by showing that the grounding of verbs in the objects they can interact with is also an important part of verbs’ semantic representation and directly influences lexical retrieval speed. This general trend seems to illustrate that understanding verb meaning through semantic richness effects is a notable aspect when investigating how verb processing relates to the semantic information typically assumed to affect processing on nouns.

As PPI shows similar effects to other variables within the grounded cognition framework, it is important to consider what is unique about PPI. In contrast to RE that is a measure of the degree of body involvement a verb evokes, PPI measures the number of potential physical interactions a verb can have. These two measures assess different properties of the semantic representation of verbs. First, we note that the correlation between RE and PPI is quite low (r =.190). This is based on only 63 of our stimuli because the RE ratings do not exist for the remaining verbs. Second, comparing a couple of the words that have ratings for both measures shows how the two diverge. A word like *tickle* rates high on RE but low on PPI. This seems appropriate as the human body can definitely experience being tickled, but the potential physical interactions are small. For a word like *submit*, RE is low, but PPI is high. The list of things that can be submitted is large, but there is not much body involvement. Based on the low correlation and the fact that the two measures try to quantify different dimensions of verb information, we believe that PPI and RE are distinct constructs and are both needed to understand the semantic representation of action verbs. Likewise, we believe that PPI provides unique information relative to BOI that is also rooted within the grounded cognition framework. BOI was designed to measure how easily an object can be acted on by the human body (see BOI norming instructions in Pexman et al., 2019). The PPI measure goes in the other direction and asks how many objects a verb can interact with. BOI is concerned with the semantic representation of objects whereas PPI is concerned with the semantic representation of verbs. For these reasons, we believe that PPI provides a novel and potentially useful measure of the semantic representation of action verbs.

Given that verbs have not been investigated as much as nouns with regards to semantic processes, the current findings show that future research should consider and approach verb processing through grounded information and potential semantic richness effects. Given that semantic richness relies on information density and that verbs seemingly rely on motor information, other aspects that integrate grounded information related to verb stimuli should be considered. Overall, grounded information plays a role in the lexical processing of verbs, and in line with the hypothesis of semantic richness, more grounding means a richer semantic representation.

## Supplementary Information

Below is the link to the electronic supplementary material.

Supplementary file1 (DOCX 23 KB)

## Data Availability

The data are available at https://osf.io/tkan5/?view_only=7d1674b18f704aa282707f44193f4104. The experiment was not preregistered.

## Footnotes

1 We have also included an analysis on the OSF site that runs the LMM with all the variables in Table 3 and two additional control variables, semantic diversity (Hoffman et al., 2013) and sensorimotor strength (Lynott et al., 2020) to guard against any potential confounding from these variables. The results show that PPI is significant, p <.001, and neither semantic diversity nor sensorimotor strength were significant, both p >.40.

## Code availability

The R Markdown file containing all model analyses is available at https://osf.io/tkan5/?view_only=7d1674b18f704aa282707f44193f4104.

## References

[CR1] Amsel, B. D., Urbach, T. P., & Kutas, M. (2012). Perceptual and motor attribute ratings for 559 object concepts. *Behavior Research Methods,**44*(4), 1028–1041. 10.3758/s13428-012-0215-z 22729692 10.3758/s13428-012-0215-z PMC3480996

[CR2] Baayen, R. H., & Milin, P. (2010). Analyzing reaction times. *International Journal of Psychological Research,**3*(2), 12–28.

[CR3] Balota, D. A., Yap, M. J., Hutchison, K. A., Cortese, M. J., Kessler, B., Loftis, B., Neely, J. H., Nelson, D. L., Simpson, G. B., & Treiman, R. (2007). The english lexicon project. *Behavior Research Methods,**39*(3), 445–459. 10.3758/BF03193014 17958156 10.3758/bf03193014

[CR4] Barsalou, L. W. (1999). Perceptual symbol systems. *Behavioral and Brain Sciences,**22*(4), 577–660. 11301525 10.1017/s0140525x99002149

[CR5] Barsalou, L. W. (2008). Grounded cognition. *Annual Review of Psychology,**59*, 617–645. 17705682 10.1146/annurev.psych.59.103006.093639

[CR6] Barsalou, L. W., Kyle Simmons, W., Barbey, A. K., & Wilson, C. D. (2003). Grounding conceptual knowledge in modality-specific systems. *Trends in Cognitive Sciences,**7*(2), 84–91. 10.1016/S1364-6613(02)00029-3 12584027 10.1016/s1364-6613(02)00029-3

[CR7] Bates, D., Maechler, M., Bolker, B., & Walker, S. (2023). *lme4 (version 1.1-35.5) [computer software]*. CRAN. https://cran.r-project.org/web/packages/lme4

[CR8] Bennett, S. D. R., Burnett, A. N., Siakaluk, P. D., & Pexman, P. M. (2011). Imageability and body–object interaction ratings for 599 multisyllabic nouns. *Behavior Research Methods,**43*(4), 1100–1109. 10.3758/s13428-011-0117-5 21681627 10.3758/s13428-011-0117-5

[CR9] Buchanan, L., Westbury, C., & Burgess, C. (2001). Characterizing semantic space: Neighborhood effects in word recognition. *Psychonomic Bulletin & Review,**8*(3), 531–544. 10.3758/BF03196189 11700905 10.3758/bf03196189

[CR10] Cortese, M. J., Yates, M., Schock, J., & Vilks, L. (2018). Examining word processing via a megastudy of conditional reading aloud. *Quarterly Journal of Experimental Psychology*. 10.1177/1747021817741269 10.1177/1747021817741269 30362414

[CR11] Glenberg, A. M. (2015). Few believe the world is flat: How embodiment is changing the scientific understanding of cognition. *Canadian Journal of Experimental Psychology = Revue Canadienne De Psychologie Experimentale,**69*, 165–171. 10.1037/cep0000056 26010024 10.1037/cep0000056

[CR12] Hoffman, P., Lambon Ralph, M. A., & Rogers, T. T. (2013). Semantic diversity: A measure of semantic ambiguity based on variability in the contextual usage of words. *Behavior Research Methods,**45*(3), 718–730. 10.3758/s13428-012-0278-x 23239067 10.3758/s13428-012-0278-x

[CR13] Hoffman, P., & Woollams, A. M. (2015). Opposing effects of semantic diversity in lexical and semantic relatedness decisions. *Journal of Experimental Psychology: Human Perception and Performance,**41*(2), 385. 10.1037/a0038995 25751041 10.1037/a0038995 PMC4378535

[CR14] Kuznetsova, A., Brockhoff, P. B., Christensen, R. H. B., & Jensen, S. P. (2020). lmerTest (version 3.1-3) [computer software]. CRAN. https://cran.r-project.org/web/packages/lmerTest/

[CR15] Lakoff, G. (2012). Explaining embodied cognition results. *Topics in Cognitive Science,**4*(4), 773–785. 10.1111/j.1756-8765.2012.01222.x 22961950 10.1111/j.1756-8765.2012.01222.x

[CR16] Locker, L., Simpson, G. B., & Yates, M. (2003). Semantic neighborhood effects on the recognition of ambiguous words. *Memory & Cognition,**31*(4), 505–515. 10.3758/BF03196092 12872867 10.3758/bf03196092

[CR17] Lynott, D., Connell, L., Brysbaert, M., Brand, J., & Carney, J. (2020). The Lancaster sensorimotor norms: Multidimensional measures of perceptual and action strength for 40,000 English words. *Behavior Research Methods,**52*(3), 1271–1291. 10.3758/s13428-019-01316-z 31832879 10.3758/s13428-019-01316-z PMC7280349

[CR18] McRae, K., Cree, G. S., Seidenberg, M. S., & Mcnorgan, C. (2005). Semantic feature production norms for a large set of living and nonliving things. *Behavior Research Methods,**37*(4), 547–559. 10.3758/BF03192726 16629288 10.3758/bf03192726

[CR19] Mirman, D., & Magnuson, J. S. (2008). Attractor dynamics and semantic neighborhood density: Processing is slowed by near neighbors and speeded by distant neighbors. *Journal of Experimental Psychology: Learning, Memory, and Cognition,**34*(1), 65. 18194055 10.1037/0278-7393.34.1.65 PMC2276160

[CR20] Nelson, D. L., McEvoy, C. L., & Schreiber, T. A. (2004). The University of South Florida free association, rhyme, and word fragment norms. *Behavior Research Methods, Instruments, & Computers,**36*(3), 402–407. 10.3758/BF03195588 10.3758/bf03195588 15641430

[CR21] Pexman, P. M. (2012). Meaning-based influences on visual word recognition. In J. S. Adelman (Ed.), *Visual word recognition: Meaning and context, individuals and development* (vol. 2. (2012-23042-002), pp. 24–43). Psychology Press.

[CR22] Pexman, P. M., Muraki, E., Sidhu, D. M., Siakaluk, P. D., & Yap, M. J. (2019). Quantifying sensorimotor experience: Body–object interaction ratings for more than 9,000 English words. *Behavior Research Methods,**51*(2), 453–466. 10.3758/s13428-018-1171-z 30484218 10.3758/s13428-018-1171-z

[CR23] Pexman, P. M. (2020). How does meaning come to mind? Four broad principles of semantic processing. *Canadian Journal of Experimental Psychology = Revue Canadienne De Psychologie Experimentale,**74*(4), 275–283. 10.1037/cep0000235 33104379 10.1037/cep0000235

[CR24] Pexman, P. M., Lupker, S. J., & Hino, Y. (2002). The impact of feedback semantics in visual word recognition: Number-of-features effects in lexical decision and naming tasks. *Psychonomic Bulletin & Review,**9*(3), 542–549. 10.3758/BF03196311 12412895 10.3758/bf03196311

[CR25] Pexman, P. M., Holyk, G. G., & Monfils, M.-H. (2003). Number-of-features effects and semantic processing. *Memory & Cognition,**31*(6), 842–855. 10.3758/BF03196439 14651293 10.3758/bf03196439

[CR26] Schneider, W., Eschman, A., & Zuccolotto, A. (2002). *E-prime user’s guide*. Psychology Software Tools Inc.

[CR27] Siakaluk, P. D., Pexman, P. M., Aguilera, L., Owen, W. J., & Sears, C. R. (2008). Evidence for the activation of sensorimotor information during visual word recognition: The body–object interaction effect. *Cognition,**106*(1), 433–443. 10.1016/j.cognition.2006.12.011 17258186 10.1016/j.cognition.2006.12.011

[CR28] Sidhu, D. M., Kwan, R., Pexman, P. M., & Siakaluk, P. D. (2014). Effects of relative embodiment in lexical and semantic processing of verbs. *Acta Psychologica,**149*, 32–39. 10.1016/j.actpsy.2014.02.009 24657828 10.1016/j.actpsy.2014.02.009

[CR29] Yap, M., Pexman, P., Wellsby, M., Hargreaves, I., & Huff, M. (2012). An abundance of riches: Cross-task comparisons of semantic richness effects in visual word recognition. *Frontiers in Human Neuroscience*, *6*. 10.3389/fnhum.2012.00072 10.3389/fnhum.2012.00072 PMC3328122 22529787

[CR30] Yap, M., Lim, G. Y., & Pexman, P. M. (2015). Semantic richness effects in lexical decision: The role of feedback. *Memory & Cognition,**43*(8), 1148–1167. 10.3758/s13421-015-0536-0 26155967 10.3758/s13421-015-0536-0

