# Supplementary material for: Grounding verbs in action: The facilitative effect of potential physical interactions with verbs
Source: Psychon Bull Rev. 2026 Jan 5;33(1):34. doi: 10.3758/s13423-025-02776-5 (PMC12769990; doi:10.3758/s13423-025-02776-5)
Supplement: Supplementary file 1 — Supplementary file1 (DOCX 23 KB) [file 13423_2025_2776_MOESM1_ESM.docx]

Appendix

In the following survey, you will be given a set of action verbs to evaluate on a scale based on the number of “physical interactions” it can potentially have, from few to many. This means as a verb is presented to you, you should think of how it rates in relation to this measure on the scale we present. To define more precisely, the measurement of “physical interactions” would indicate anything to which the verb can reasonably be used upon in the most concrete sense, such as to ***eat****an****apple***, or *to****stretch****a****cloth*** (however, *to****eat****your own****words*** or *to****stretch****yourself****thin*** would not be a physical interaction for the verb ***eat*** or ***stretch*** because it is used in the abstract sense). For example, on this scale, the action verb ***tap*** would rank many due to the common nature of the action and its potential interactions. The action verb ***read***, however, would rank few on this scale due to the limited number of potential physical interactions that the verb can be used upon.

Rating Scale

| Few |  |  |  |  |  | Many |
| --- | --- | --- | --- | --- | --- | --- |
| 1 | 2 | 3 | 4 | 5 | 6 | 7 |
